# Prediction of left ventricular reverse remodeling after therapy with angiotensin-converting enzyme inhibitors or angiotensin II receptor blockers and β blockers in patients with idiopathic dilated cardiomyopathy

**DOI:** 10.1186/s12947-015-0009-4

**Published:** 2015-03-25

**Authors:** Yoshihisa Matsumura, Eri Hoshikawa-Nagai, Toru Kubo, Naohito Yamasaki, Hiroaki Kitaoka, Jun Takata, Yoshinori Doi, Tetsuro Sugiura

**Affiliations:** Department of Laboratory Medicine, Kochi Medical School, Kochi University, Nankoku-shi, Kochi, Oko-cho 783-8505 Japan; Department of Cardiology, Neurology, and Aging Science, Kochi Medical School, Kochi University, Kochi, Japan; Center to Promote Creativity in Medical Education, Kochi Medical School, Kochi University, Kochi, Japan; Chikamori Hospital, Kochi, Japan

**Keywords:** Remodeling, Atrial fibrillation, Cardiomyopathy, Heart failure

## Abstract

**Background:**

Predictors of left ventricular reverse remodeling (LVRR) after therapy with angiotensin converting enzyme inhibitors or angiotensin-receptor blockers and β blockers in patients with idiopathic dilated cardiomyopathy (IDC) remains unclear.

**Methods:**

We studied 44 patients with IDC who had been treated with the therapy. LVRR was defined as LV end-diastolic dimension ≤ 55 mm and fractional shortening ≥ 25% at the last echocardiogram.

**Results:**

During a mean follow-up period of 4.7 ± 3.3 years, LVRR occurred in 34% (15/44) of the patients. We divided the patients into 2 groups: (1) patients with LVRR (n = 15); (2) patients without LVRR (n = 29). The presence of atrial fibrillation was 40% in patients with LVRR and 14% in those without (p = 0.067). Initial LV end-diastolic dimension was significantly smaller (62 ± 6 vs. 67 ± 6 mm, p = 0.033) in patients with LVRR than in those without. Initial LV end-diastolic dimension of 63.5 mm was an optimal cutoff value for predicting LVRR (sensitivity: 67%, specificity: 59%, area under the curve: 0.70, p = 0.030). When patients were further allocated according to initial LV end-diastolic dimension ≤ 63.5 mm with atrial fibrillation, the combined parameter was a significant predictor of LVRR by univariate logistic regression analysis (odds ratio, 5.78, p = 0.030) (sensitivity: 33%, specificity: 97%, p = 0.013).

**Conclusions:**

Combined information on LV end-diastolic dimension and heart rhythm at diagnosis is useful in predicting future LVRR in patients with IDC.

## Introduction

Idiopathic dilated cardiomyopathy (IDC) is characterized by left ventricular (LV) dilatation with systolic dysfunction [1]. Reverse remodeling (RR), which is a decrease in LV size with an improvement in systolic function, has an important role in prognosis of IDC [2-10]. Recently, occurrence of LVRR during follow-up has been reported to identify patients who will have a favorable future prognosis [5,8]. Therefore, prediction of future LVRR at initial diagnosis is of prognostic significance. Nevertheless, predictors of LVRR remain unclear in IDC [11]. The aim of the present study was to identify predictors of LVRR in patients with IDC after therapy with angiotensin converting enzyme (ACE) inhibitors or angiotensin-receptor blockers (ARBs) and β blockers.

## Methods

We retrospectively studied 44 patients with IDC who were treated with therapy with ACE inhibitors or ARBs and β blockers. ACE inhibitors or ARBs and β blockers were continued during follow-up in all patients, although there were some changes of the other concomitant drugs, such as diuretics, when clinically indicated. All patients were admitted to our hospital for confirmation of diagnosis, risk assessment, and symptom management during the period from 1994 to 2006. The study was approved by the Ethics Committee on Medical Research of the Kochi Medical School. All patients gave informed consent. On admission, an exhaustive clinical evaluation including medical history, physical examination, 12-lead electrocardiography, ambulatory 24-hour electrocardiography, laboratory studies, echocardiography, and cardiac catheterization was performed, in each patient to identify cause of cardiomyopathy as precisely as possible. The diagnostic criteria were: (1) dilated LV end-diastolic dimension (Dd) > 55 mm with fractional shortening (FS) < 25%; (2) exclusion of patients with acute myocarditis, infiltrative myocardial disease, connective-tissue disease, endocrine dysfunction, neuromuscular disease, general systemic disease, significant coronary artery stenosis (defined as diameter narrowing of > 50% in any of the major coronary arteries or their branches), valvular disease, sensitivity/toxic reactions and a history of excessive alcohol intake. LVDd, LV end-systolic dimension (Ds), thicknesses of the interventricular septum, LV posterior wall, and left atrial dimension were measured by M-mode echocardiography as recommended by the American Society of Echocardiography [12]. LVFS was calculated as ((LVDd – LVDs)/LVDd) × 100. Echocardiography was performed in routine clinical practice. The study patients underwent echocardiography at baseline and within 1 year of the last visit, death, or transplantation. LV reverse remodeling (LVRR) was defined as described previously (LV end-diastolic dimension (Dd) ≤ 55 mm and fractional shortening (FS) ≥ 25% at the last echocardiogram) [5,10]. Follow-up data were obtained by regular visits and chart reviews, and telephone contact with the patients or their relatives.

### Statistical analysis

Categorical variables are presented as total number and % of patients, and continuous variables are presented as means ± standard deviation. Fisher’s exact test was used to analyze categorical variables. Differences in continuous variables were analyzed by the unpaired Student’s *t* test or Mann–Whitney test, as appropriate. Receiver operating characteristic curve analysis was used to determine the discriminating cutoff value for predicting LVRR. Univariate logistic regression analysis was used to determine a significant predictor of LVRR. A p value of < 0.05 was considered statistically significant.

## Results

**Figure 1:**
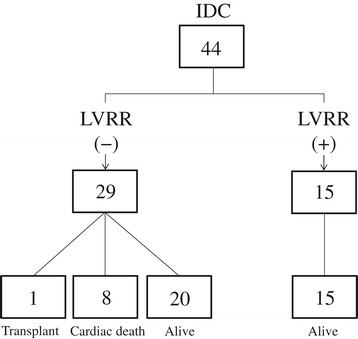
**Occurrence of LVRR and clinical outcomes during a follow-up period of 4.7 ± 3.3 years.** LVRR, left ventricular reverse remodeling; IDC, dilated cardiomyopathy.

The incidence of LVRR and clinical outcomes during a mean follow-up period of 4.7 ± 3.3 years (range 5 months to 12 years) are shown in Figure 1. LVRR occurred in 34% (15/44) of the patients. LVRR occurred at 6 months in 2 patients, and after 12 months in 13 patients. All patients who showed LVRR survived. Of the remaining 29 patients without LVRR, 8 patients died (heart failure death in 5 patients, sudden cardiac death in 3), 1 underwent heart transplantation, and 20 survived. The incidence of cardiac death and heart transplantation was significantly higher in patients without LVRR than in those without (p = 0.018).

We divided the patients into 2 groups: (1) patients with LVRR, (2) patients without LVRR. There were no significant differences in the frequency of use of ACE inhibitors or ARBs. We most frequently used enalapril (83%) (30/36) as an ACE inhibitor and losartan (63%) (5/8) as an ARBs. There were no significant differences in these maintenance doses between the 2 groups. Carvedilol was administered in 37 patients and metoprolol in 7 patients. There were no significant differences in the frequency of use of these drugs. There were no significant differences in these maintenance doses between the 2 groups (Table 1).

**Table 1:** **Initial clinical characteristics**

**Variables**	**LVRR (+)**	**LVRR (−)**	**p value**
	**(n = 15)**	**(n = 29)**	
Age (years)	60 ± 11	58 ± 13	0.512
Men	13 (87%)	26 (89%)	0.767
New York Heart Association class			
I – II	11	24	0.207
III – IV	5	5	
Diabetes mellitus	4 (27%)	3 (10%)	0.206
Atrial fibrillation	6 (40%)	4 (14%)	0.067
Nonsustained ventricular tachycardia	6 (40%)	11 (38%)	0.894
Serum creatinine (mg/dl)	0.87 ± 0.16	0.81 ± 0.25	0.406
Estimated glomerular filtration rate (ml min^-1^ 1.73 m^-2^)	80.3 ± 12.2	79.8 ± 12.1	0.738
Complete left bundle brunch brock	2 (13%)	6 (21%)	0.549
QRS duration (ms)	101 ± 14	111 ± 32	0.173
Follow-up periods (years)	5.9 ± 3.2	4.4 ± 2.8	0.220
Pharmacological treatments			
β blockers	15 (100%)	29 (100%)	>0.99
Carvedilol	13 (87%)	24 (83%)	0.737
Dose (mg/day)	11.3 ± 4.8	10.8 ± 5.3	0.761
Metoprolol	2 (13%)	5 (17%)	0.735
Dose (mg/day)	60.0 ± 28.2	56.0 ± 21.9	0.879
Angiotensin converting enzyme inhibitors/angiotensin II receptor blockers	12 / 3 (100%)	24/5 (100%)	>0.99
Enalapril	10 (67%)	20 (69%)	0.877
Dose (mg/day)	5.2 ± 1.8	4.6 ± 0.9	0.318
Losartan	2 (13%)	3 (20%)	0.767
Dose (mg/day)	37.5 ± 17.7	41.7 ± 14.4	0.738
Loop diuretics	13 (87%)	27 (93%)	0.596
Spironolactone	7 (45%)	15 (52%)	0.751
Digitalis	11 (73%)’	20 (67%)	0.763
Amiodarone	1 (7%)	4 (14%)	0.647

Data are presented as mean ± SD or n (%). LVRR, left ventricular reverse remodeling.

Atrial fibrillation was found in 40% (6/15) of patients with LVRR, and in 14% (4/29) of those without LVRR (p = 0.067). The initial heart rate was 87 ± 21 (60–105) beats/min in 6 patients with LVRR, and that was 98 ± 28 (80–140) beats/min in 4 patients without LVRR. No difference was found in the initial heart rate between the 2 groups (P = 0.390). The heart rate was > 100 beats/min was found in 2 patients with atrial fibrillation; 1 patient with heart rate of 105 beats/min showed LVRR, and 1 patient with heart rate of 140 beats/min did not show LVRR. Atrial fibrillation recovered to sinus rhythm in 2 patients who did not show LVRR. Initial LVDd was significantly smaller in patients with LVRR than in those without LVRR (Table 2). No other differences were found between the 2 groups. Initial and last echocardiographic parameters are shown in Table 3. Initial LVDd of 63.5 mm was an optimal cutoff value for predicting LVRR (sensitivity: 67%, specificity: 59%, area under the curve: 0.70, p = 0.030) by receiver operating characteristic curve analysis. When patients were further allocated according to initial LVDd ≤ 63.5 mm in combination with atrial fibrillation, initial LVDd ≤ 63.5 mm with atrial fibrillation was a significant predictor of LVRR by univariate logistic regression analysis (odds ratio, 5.78; 95% confidence interval, 1.19 – 28.0, p = 0.030) (sensitivity: 33%, specificity: 97%, p = 0.013).

**Table 2:** **Initial echocardiographic and cardiac catheterization findings**

**Variables**	**LVRR (+)**	**LVRR (−)**	**p value**
Left ventricular end-diastolic dimension (mm)	62 ± 6	67 ± 6	0.033
Left ventricular end-systolic dimension (mm)	53 ± 6	57 ± 8	0.093
Left ventricular fractional shortening (%)	15 ± 4	14 ± 5	0.574
Interventricular septal thickness (mm)	10 ± 2	10 ± 1	0.727
Left ventricular posterior wall thickness (mm)	10 ± 2	9 ± 2	0.165
Relative wall thickness	0.32 ± 0.01	0.29 ± 0.06	0.106
Left atrial dimension (mm)	43 ± 6	42 ± 7	0.653
Left ventricular mass index (g/m^2^)	204 ± 58	196 ± 68	0.703
Left ventricular end-diastolic volume index (ml/m^2^)	144 ± 72	169 ± 43	0.197
Left ventricular end-systolic volume index (ml/m^2^)	100 ± 66	119 ± 42	0.274
Left ventricular ejection fraction (%)	34 ± 13	31 ± 9	0.357
Left ventricular end-diastolic pressure (mm Hg)	12 ± 6	12 ± 7	0.819
Pulmonary capillary wedge pressure (mm Hg)	11 ± 8	11 ± 8	0.929
Systolic pulmonary artery pressure (mm Hg)	30 ± 12	29 ± 9	0.672
Mean pulmonary artery pressure (mm Hg)	19 ± 8	19 ± 9	0.961
Right ventricular end-diastolic pressure (mm Hg)	8 ± 3	7 ± 4	0.806
Mean right atrial pressure (mm Hg)	6 ± 2	6 ± 4	0.963
Systolic aortic pressure (mm Hg)	112 ± 22	112 ± 19	0.985
Mean aortic pressure (mm Hg)	87 ± 15	84 ± 12	0.614
Cardiac index (ml/min/m^2^)	2.1 ± 0.6	2.2 ± 0.6	0.486

Data are presented as mean ± SD. LVRR, left ventricular reverse remodeling.

**Table 3:** **Initial and last echocardiographic findings**

**Variables**	**LVRR (+)**		**LVRR (−)**	
	**Initial**	**Last**	**Initial**	**Last**
Left ventricular end-diastolic dimension (mm)	62 ± 6	49 ± 4	67 ± 6	62 ± 9
Left ventricular end-systolic dimension (mm)	53 ± 6	33 ± 4	57 ± 8	50 ± 11
Left ventricular fractional shortening (%)	15 ± 4	32 ± 4	14 ± 5	20 ± 8
Interventricular septal thickness (mm)	10 ± 2	10 ± 1	10 ± 1	10 ± 1
Left ventricular posterior wall thickness (mm)	10 ± 2	10 ± 1	9 ± 2	9 ± 1
Relative wall thickness	0.32 ± 0.01	0.41 ± 0.06	0.29 ± 0.06	0.30 ± 0.08
Left atrial dimension (mm)	43 ± 6	42 ± 6	42 ± 7	41 ± 7
Left ventricular mass index (g/m 2)	204 ± 58	140 ± 30	196 ± 68	176 ± 54

Data are presented as mean ± SD. LVRR, left ventricular reverse remodeling.

## Discussion

The present study had major 2 findings. First, initial LVDd was significantly smaller in patients with LVRR than in those without. Second, when patients were further allocated according to initial LV end-diastolic dimension ≤ 63.5 mm with atrial fibrillation, the combined parameter was a significant predictor of LVRR by univariate logistic regression analysis (odds ratio: 5.78, p = 0.030).

LVRR has a key role in favorable prognosis of IDC [2-10]. Although many predictors of LVRR in patients with IDC have been reported, inconsistent results exist in the past studies [2,8,11,13-19]. This was probably because of differences in the definition of LVRR and in clinical factors such as pharmacological therapy. Although the ACE inhibitors or ARBs and β blockers that block the neurohormonal activation play an important role in inducing LVRR, there is no report on prediction of LVRR after therapy with ACE inhibitors or ARBs and β blockers in patients with IDC. In the present study, initial LVDd was smaller in patients with LVRR than in those without LVRR. Initial LVDd of ≤ 63.5 mm was significantly associated with future LVRR by receiver operating characteristic curve analysis. In a past study, myocardial recovery was evident in 32% of the patients on a LV assist device who had initial LVDd < 60 mm [20]. In contrast, myocardial recovery was not evident in all patients who had initial LVDd > 70 mm. More recently, in the multicenter IMAC-2 study, LVDd at presentation predicted a better LV systolic function at 6 months [21]. The authors have stated that smaller LV size is likely a marker of a more reversible cardiac pathological condition. Similarly, the present study suggests that initial LVDd could provide important information in predicting future LVRR.

Atrial fibrillation is a common arrhythmia in patients with IDC. The presence of atrial fibrillation tended to be associated with LVRR in the present study. When patients were further categorized according to initial LVDd ≤ 63.5 mm with concomitant atrial fibrillation, this combined parameter was a significant predictor of LVRR by univariate logistic regression analysis. The parameter of initial LVDd ≤ 63.5 mm with concomitant atrial fibrillation had high specificity and low sensitivity. These results suggest that the combined parameter is useful for predicting future LVRR, but not useful for denying future LVRR.

It is problematic to determine whether atrial fibrillation is the primary cause of the cardiomyopathy (tachycardia-induced cardiomyopathy), or secondary to IDC [22,23]. We are still in this old dilemma of “which came first”: chicken, or egg [24]? Tachycardia-induced cardiomyopathy is retrospectively diagnosed by marked improvement in LV function typically seen in 4 – 6 weeks [23]. Prolonged heart rate > 100 beats/min has been reported to be also important in its diagnosis [23]. However, there are no absolute parameters which distinguish between tachycardia-induced cardiomyopathy and IDC. In the present study, the patients with atrial fibrillation had not typical feature of tachycardia-induced cardiomyopathy in view of the initial heart rate and time of appearance of LVRR. Also, no significant difference was found in initial LVDd between patients with atrial fibrillation and those without (data not shown). Although these results indicate that patients of the present study with atrial fibrillation had IDC but not tachycardia-induced cardiomyopathy, initial LV end-diastolic dimension ≤ 63.5 mm with atrial fibrillation was a significant predictor of LVRR, suggesting that atrial fibrillation might be associated with future LVRR.

The targeting doses of ACE inhibitors, ARBs, and β blockers were lower in the present study than those in the United States’ guidelines [25]. A low dose of carvedilol of 5 mg/day was beneficial in Japanese patients with heart failure in the Multicenter Carvedilol Heart Failure Dose Assessment (MUCHA) trial [26]. We have previously reported that low doses of ACE inhibitors, ARBs, and β blockers had favorable effects on the prognosis of Japanese patients with IDC [27,28]. The Japanese Guidelines (available at the Japanese Circulation Society Web site (http://www.j-circ.or.jp/) have recommended a targeting dose of enalapril of 5 to 10 mg/day and of carvedilol of 5 to 20 mg/day.

The present study has several limitations as follows: (1) The study was retrospective, and the number of patients was small; (2) Although all patients showed basically diffuse LV wall motion abnormalities, calculated LVFS would not be a representative estimate of systolic function, particularly when regional abnormalities were present; (3) Because these limitations could affect the results of the present study, care should be taken when applying the results to the individual patients; (4) There are no currently available parameters that can accurately distinguish between tachycardia-induced cardiomyopathy and IDC; (5) Further studies especially with a large number of patients are required to confirm the results of the present study.

## Conclusions

Initial LVDd was significantly smaller in patients with LVRR than in those without. Initial LVDd ≤ 63.5 mm in combination with atrial fibrillation was a significant predictor of future LVRR. Combined information on LVDd and heart rhythm at diagnosis is useful in predicting future LVRR in patients with IDC.

## Abbreviations

LV: Left ventricular
RR: Reverse remodeling
IDC: Idiopathic dilated cardiomyopathy
ACE: Angiotensin converting enzyme
ARBs: Angiotensin-receptor blockers
Dd: End-diastolic dimension
Ds: End-systolic dimension
FS: Fractional shortening
